# The Immune Landscape of Chinese Head and Neck Adenoid Cystic Carcinoma and Clinical Implication

**DOI:** 10.3389/fimmu.2021.618367

**Published:** 2021-09-06

**Authors:** Shengjin Dou, Rongrong Li, Ning He, Menghuan Zhang, Wen Jiang, Lulu Ye, Yining Yang, Guodong Zhao, Yadong Yang, Jiang Li, Di Chen, Guopei Zhu

**Affiliations:** ^1^Radiotherapy Division, Department of Oral and Maxillofacial-Head Neck Oncology, Shanghai Ninth People’s Hospital, College of Stomatology, Shanghai Jiao Tong University School of Medicine, Shanghai, China; ^2^National Center for Stomatology and National Clinical Research Center for Oral Diseases, Shanghai, China; ^3^Shanghai Key Laboratory of Stomatology, Shanghai, China; ^4^GloriousMed Technology Co., Ltd, Shanghai, China; ^5^Department of Oral Pathology, Shanghai Ninth People’s Hospital, College of Stomatology, Shanghai Jiao Tong University School of Medicine, Shanghai, China

**Keywords:** immune infiltration, tumor microenvironment (TME), immune checkpoint inhibitors (ICIs), PD-1/PD-L1, adenoid cystic carcinoma (ACC)

## Abstract

Novel systemic agents and effective treatment strategies for recurrence adenoid cystic carcinoma (ACC) of the head and neck are still worthy of further exploration. Here, we analyzed the mutations and expression profiles of 75 Chinese ACC patients, characterized the prognostic value of the immune signature for recurrence or distant metastasis, and explored the potential of immunotherapeutic biomarkers in ACC. In general, MYB fusion and somatic mutations accounted for a high proportion, which was 46.7% (35/75). ACCs displayed an overall low mutation burden and lack of programmed cell death ligand-1 (PD-L1) expression. The antigen-presenting machinery (APM) expression score and immune infiltration score (IIS) were the lowest among ACC patients, compared with other cancer types. For 61 primary cases, the locoregional recurrence-free survival (LRRFS) was statistically significantly correlated with the IIS [univariate analysis; hazard ratio (HR) = 0.32; 95% CI, 0.11–0.92; p = 0.035] and T-cell infiltration score (TIS) (univariate analysis; HR = 0.33; 95% CI, 0.12–0.94; p = 0.037]. Patients with lower IIS (log-rank p = 0.0079) or TIS (log-rank p = 0.0079) had shorter LRRFS. Additionally, solid pattern was also a prognostic factor related to locoregional recurrence, whereas postoperative radiotherapy (PORT) exerted its beneficial effects. We further evaluated the pretreatment immune profile of five ACC patients treated with PD-1 inhibitors. Patients who responded to camrelizumab or pembrolizumab observed elevated APM and TIS, compared with patients with progressive disease. Our study highlights the immune infiltration pattern and messenger RNA (mRNA) signatures of Chinese ACC patients, which has the potential value for prognosis and immunotherapy.

## Introduction

Adenoid cystic carcinoma (ACC) is a rare malignancy predominantly arising from salivary glands, accounting for about 1% of all head and neck malignant tumors (1–3). ACC is characterized by indolent but relentless growing, perineural invasion and perineural spread, high propensity for local recurrence after initial treatment, and common distant metastasis (4). Although postoperative radiotherapy has been shown to increase the local control rate by 89–95% within 5 years (5, 6), the disease-free survival rates decline dramatically at 10 and 15 years (6–13). Therefore, novel systemic agent and effective treatment strategy for recurrence ACC are imperative to explore.

Immunotherapy is an important component of cancer treatment, especially recent advances in immune checkpoint inhibitors (ICIs) have begun to transform clinical cancer care (14). As one of the most successful immunotherapies, ICIs has been approved in a variety of solid tumor types. However, despite immune checkpoint therapy has demonstrated remarkable clinical efficacy in subsets of patients, the majority of patients did not show durable responses (15–17). Therefore, to better understand and overcome the mechanism of resistance, increasing studies have focused on the identification and development of predictive biomarkers of ICI response.

Selected biomarkers involving tumor mutational burden (TMB), microsatellite instability (MSI), programmed cell death ligand-1 (PD-L1) expression, and tumor-infiltrating lymphocytes have shown early promise in predicting response and benefit from ICIs (14, 18). Emerging data suggest that the tumor microenvironment (TME) may be a promising predictive biomarker for the survival benefit and prognosis of immunotherapy (19, 20). TME is complex and continuously evolving, which contains extracellular matrix and diverse cell types, such as fibroblasts, adipose cells, tissue-resident and peripherally recruited immune cells, and endothelial cells (21). Previous researches have suggested that immune cells in TME, as regulators in cancer progression, are becoming alluring therapeutic targets (21–24). For instance, tumor associated macrophages (TAMs) and regulatory T cells (Tregs) have been regarded to be protumor (25, 26), while CD8^+^ T cells are associated with improved clinical outcomes and response to immunotherapy (27–29). The proliferation and activation of CD8^+^ T cell rely on their T-cell receptor (TCR) recognizing the peptide antigen presented by major histocompatibility class I (MHC-I) on a target cell, evoking an antigen-specific immune response, thereby killing antigen-bearing cells (30). Antigen-presenting machinery (APM) genes encodes MHC-I subunits and proteins, which are essential for processing antigens and burden them onto MHC-I. Activated CD8^+^ T cells and other immune infiltrates can secrete type II interferon gamma (IFN-γ), which induces upregulation of APM genes (31). Although the identification of CD8^+^ T cells may be a predictive biomarker of response to immunotherapy in some contexts (32), it is not adequate to depict the cytotoxic potential of the complex TME.

**Figure 1:**
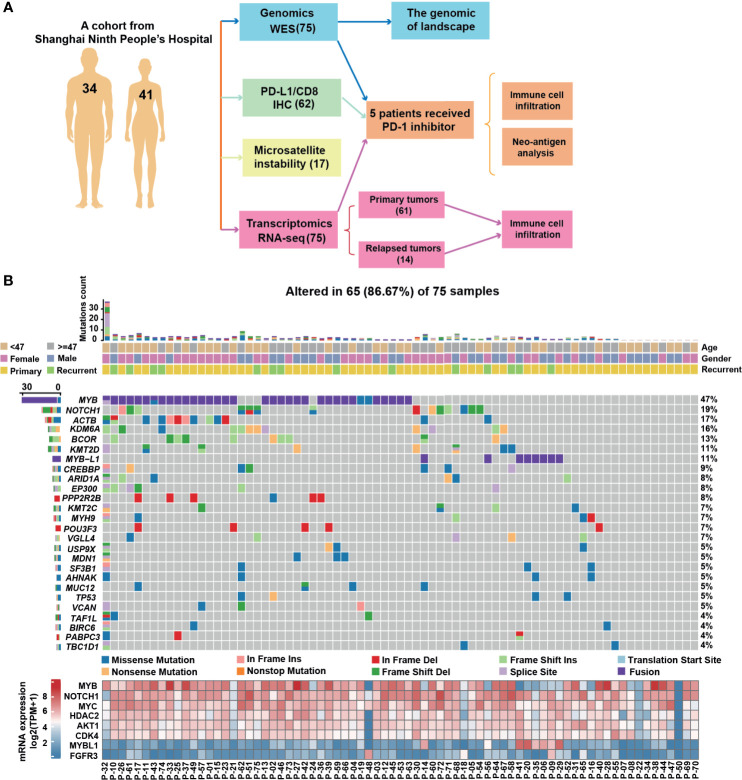
The genomic landscape of adenoid cystic carcinoma. **(A)** Workflow of genetic hallmarks and immune-infiltrate profiling in ACC patients. **(B)** Mutation rate and type, age, gender, and surgical outcomes. Ten tumor samples were undetectable of any variant with allele frequencies (AFs) ≥1%. Bottom panel, RNA expression level for selected genes, expressed as log_2_(TPM + 1) for all samples. TPM, transcripts per million (TPM) expression value.

To date, the molecular mechanism underlying the oncogenic activity of molecular alterations and tumor immune microenvironment in ACC remain elusive. We propose that ACCs own a distinct immune landscape, which might indicate diverse prognoses and treatment responses. Here, we first evaluate the genomic characteristics and the biomarkers currently used for checkpoint immunotherapy in 75 ACC cohort (**Figure 1A**). Then, we employed an APM score, a T-cell infiltration score (TIS), and an overall immune infiltration score (IIS) to highlight the immune infiltration status and their correlation with pathological features. Furthermore, the abundance of immune cells in TME of ACC was analyzed. Finally, in a small series of patients receiving anti-PD-1 agents, we assessed the correlation between immune signatures and the response to checkpoint blockade therapy. This study integrated and analyzed the whole exome, whole transcriptome, and clinical data to improve the understanding of TME in ACC.

## Methods

### Clinical Sampling and Processing

We performed a retrospective study on patients with ACC. The study was conducted in accordance with the International Ethical Guidelines for Biomedical Research Involving Human Subjects (CIOMS) and was approved by the Ethics Committee of Shanghai Ninth People’s Hospital (approval number: 2016-74-T31). Additionally, a single-center database project involving head and neck cancer was approved in 2020 (approval number: SH9H-2020-T58-1), providing us with partial patient data. An independent cohort of 75 patients with head and neck ACC were included. They enrolled into Shanghai Ninth People’s Hospital from 2012 to 2019. All participants obtained written informed consent. The main criteria were as follows: (a) informed consent; (b) no comorbidities (e.g., had suffered from other malignant tumors.); (c) complete and usable follow-up data; (d) a radical surgery performed and postoperative histopathology diagnosis confirmed; and (f) tumor stage classification was carried out according to the 7th edition of the American Joint Committee on Cancer TNM staging system. Pathological data consisted of tumor and nodal stage and subtype according to the 2015 World Health Organization (WHO) classification. Locoregional recurrence-free survival (LRRFS) was calculated from the day of surgery to first local–regional recurrence or death from any cause. Distant metastasis-free survival time (DMFS) was calculated from the day of surgery to the first distant metastases or death from any cause, identified by physical examination, positron-emission tomography–computed tomography (PET-CT) or CT or the most recent follow-up. We ended follow-up in February 2021. The median follow-up time for this cohort was 40.3 months. The 3-year rate LRRFS and DMFS rate in 61 primary cases were 27.9% (17/61) and 49.2% (30/61), respectively. The objective response of five patients treated with PD-1 inhibitors therapy is evaluated according to criteria for measurable disease in Response Evaluation Criteria in Solid Tumors (RECIST). The general data of the 75 patients with ACC are shown in **Table 1**.

**Table 1:** Demographic data and clinicopathological features of the sample (N = 75).

Characteristics	Number (%)
**Age (years)**	
≥47	38 (50.7)
<47	37 (49.3)
**Gender**	
Female	41 (54.7)
Male	34 (45.3)
**Histopathology**	
Tubular	3 (4.0)
Cribriform	8 (10.7)
Solid	14 (18.7)
Mixed	36 (48.0)
AdCC *ex* PA	1 (1.3)
Unknown	13 (17.3)
**TNM stage**	
I-II	22 (29.3)
III-IV	35 (46.7)
Unknown	18 (24.0)
**Necrosis**	
Positive	15 (20.0)
Negative	43 (57.3)
Unknown	17 (22.7)
Solid subtype*	
Yes	20 (26.7)
No	42 (56.0)
Unknown	13 (17.3)
**PORT received**	
Yes	57 (76.0)
No	15 (20.0)
Unknown	3 (4.0)
**Locoregional recurrence**	
Yes	31 (41.3)
No	44 (58.7)
**Distant metastasis**	
Yes	46 (61.3)
No	29 (38.7)
**LRRFS**	
< 60 months	57 (76.0)
≥ 60 months	18 (24.0)
**DMFS**	
< 60 months	61 (81.3)
≥ 60 months	14 (18.7)

*Including presence of solid component.

AdCC ex PA, adenoid cystic carcinoma ex pleomorphic adenoma; DMFS, distant metastasis-free survival; LRRFS, locoregional recurrence-free survival; TNM, tumor-nodes-metastases; PORT, postoperative radiotherapy.

### Tumor Infiltrating Lymphocytes, PD-L1, and CD8 Immunohistochemistry

Percentages of stromal tumor-infiltrating lymphocyte (TIL) were estimated in hematoxylin and eosin sections in 62 tumor samples according to the 2014 Guidelines developed by the International TILs Working (33). Immunohistochemistry (IHC) analysis was performed on the BOND-MAX autostainer (Leica, Wetzlar, Germany) with antibodies against the following: PD-L1 (clone 22C3, pharmDx; Dako, Carpinteria, CA, USA) and CD8 (cytotoxic T cells, clone C8/144B, Celnovtebio, China). PD-L1 expression was assessed by tumor proportion score (TPS), which was defined as the percentage of tumor cells with membranous PD-L1 staining. CD8^+^ T-cell density was defined as the percentage of T cells stained with CD8 in a tumor region (central or marginal). All stained sections were independently reviewed by two pathologists. Any discrepancies were discussed together, and a consensus was achieved under the guidance of another experienced pathologist.

### Somatic Variant Calling and Filtering

Somatic mutations from whole exome sequencing data were filtered with the following rules (1): 10 allele reads support (2), allele frequency ≥5% (3), supporting reads should be below 4 in the white blood cells (WBCs) control (4), mutation frequency of tumor should be eight times higher than that of the WBC control (5), the number of mutations in PoN should not exceed 2, and (6) no significant strand bias [GATK parameter FS >60 for single-nucleotide polymorphism (SNP) and FS >200 for indel]. Variants were also functionally filtered to remove those located in non-coding regions and synonymous mutations for downstream analysis. The log2 ratio >0.6 was considered a copy gain event. The log2 ratio less than −0.7 was considered a copy loss event.

### Gene Expression Analysis

The raw RNA-sequencing reads were filtered by FastQC and aligned using the spliced read aligner STAR2.0 (34), which was supplied with the Ensembl human genome assembly (GRCh37) as the reference genome. Gene expression levels were estimated by transcripts per kilobase million (TPM). Annotations of messenger RNA (mRNA) in the human genome were retrieved from the GENCODE (v19) database (**Supplementary Table 1**). The pan-cancer raw count gene-level RNA-Seq data were downloaded from The Cancer Genome Atlas (TCGA) Data Portal (35) (https://genome-cancer.ucsc.edu/). These cohorts consisted of adrenocortical carcinoma (tumor case = 79), bladder urothelial carcinoma (BLCA, tumor case = 433), colon adenocarcinoma (COAD, tumor case = 519), kidney chromophobe (KICH, tumor case = 89), lung squamous cell carcinoma (LUSC, tumor case = 551), lung adenocarcinoma (LUAD, tumor case = 594), and skin cutaneous melanoma (SKCM, tumor case = 472). Raw count gene expression data were used for gene expression analysis.

### Gene Signatures

Marker genes that characterize immune cell types were acquired from Bindea et al. (36). As previously published by Şenbabaoğlu et al. (31), MHC class I genes (HLA-A/B/C, B2M) and genes involved in processing and loading antigens (TAP1, TAP2, and TAPBP) delineated the seven-gene APM signature; the TIS was defined as the mean of the standardized values of nine T-cell subtypes, and the overall immune infiltration score of a sample was similarly defined as the mean of the standardized values of innate and adaptive immune scores. A subset of genes from an IFN-γ gene expression signature was obtained from Efstathiou et al. (37) (**Supplementary Table 1**). Batch-corrected normalized data was input into Tumor Immune Dysfunction and Exclusion (TIDE) (38).

### Implementation of Single-Sample Gene Set Enrichment Analysis

As reported by Şenbabaoğlu et al. (31), single-sample Gene Set Enrichment Analysis (ssGSEA) was applied for quantifying immune infiltration and activity in tumors using bulk RNA-seq data. ssGSEA (39) is a rank-based method, which is implemented using R package GSVA (40). Normalized RNA-Seq or microarray data should be used as input without further processing (i.e., no standardization or log transformation).

### Statistical Analysis

One-way ANOVA using Kruskal–Wallis with Dunn’s correction for multiple comparisons were performed with GraphPad Prism 7 (GraphPad Software, CA, USA). The unsupervised clustering of tumor samples, immune cell types, and gene expression was performed with hierarchical algorithm, Ward linkage, and Euclidean distance in R. Time-to-event endpoints were estimated using the Kaplan–Meier method. Univariate and multivariate analyses were performed using Cox proportional hazards models. To be assessed in the multivariate analysis, the variable should be significant (p ≤ 0.1) in the univariate analysis. p < 0.05 were considered statistically significant (*p < 0.05).

## Results

### Patients and Tumor Characteristics

Seventy-five ACC patients were analyzed, including 61 cases of primary disease and 14 cases of recurrent disease (**Figure 1A**). Whole-exome sequencing (WES) of 75 tumors targeted 149,323 exons in 19,397 genes (mean coverage, 254×; 92.2% of target bases >50×). MuTect identified 5,330 somatic mutations, including 3,832 point mutations and 1,498 indels (insertions or deletions). The average and median somatic mutation rates were 4.55 and 0.85 per megabase (Mb), respectively. Nearly half (46.7%, 35/75) of the samples had MYB somatic alterations (**Figure 1B**), including a total of 33 ACCs with MYB fusion, which were associated with increased mRNA level. Additionally, splice sites and coding mutations involving multiple exons of MYB were also discovered. Twenty-four NOTCH1 mutations were identified in 14 tumors, and six patients harbored more than one NOTCH1 mutation. It is worth noting that we observed relatively higher levels of MYBL1 mRNA in eight tumors with MYBL1 fusions compared with negative cases. Common copy number variances (CNVs) were found in AKT1 (29 cases; 39%), FGFR3 (21 cases; 28%), HDAC2 (16 cases; 21%) and CDK4 (15 cases; 20%) in our cohort (**Supplementary Figure S1A**), which were unassociated with mRNA expression (**Figure 1B**).

### ACCs Display Overall Low Mutation Burden and PD-L1 Expression

**Figure 2:**
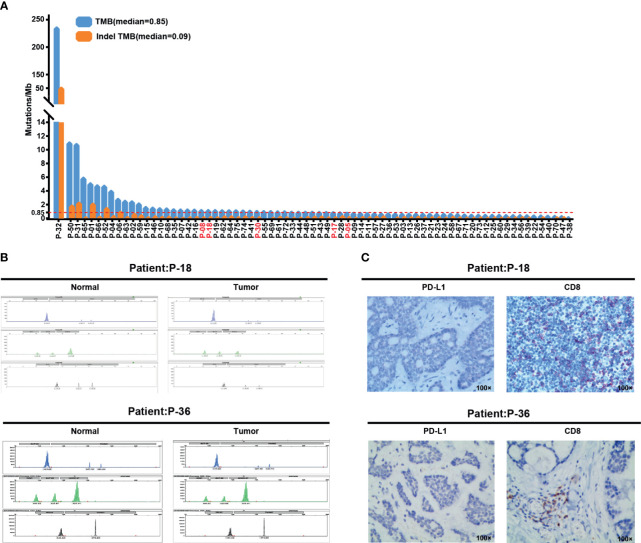
Biomarkers for checkpoint inhibitor immunotherapy in ACC patients. **(A)** Distribution of TMB and indelTMB in 75 ACC samples. The red dotted line indicates the median value of TMB. Samples are arranged in order of decreasing TMB. **(B)** Case No.P-18 and No.P-36, microsatellite stable (MSS) according to conventional MSI-polymerase chain reaction (PCR) test. **(C)** Representative images for PD-L1 and CD8 immunohistochemical staining from case No.P-18 and No. P-36 at 100× original magnification.

TMB measured by whole-exome sequencing is associated with clinical benefit of multiple checkpoint inhibitors (41). TMB is defined as the number of non-synonymous mutations per 1 Mbp and divided into tertiles, while indelTMB is composed of small insertions and deletions with frameshifts. The median TMB was 0.85 Muts/Mbp (0–230.33 Muts/Mbp), and median indelTMB was 0.09 Muts/Mbp (0–49.64 Muts/Mbp, **Figure 2A**). Except for case No.P-32 who harbored numerous hotspot mutations (**Figure 1B** and **Supplementary Table 4**), the presented cases exhibit a low mutation burden, which is consistent with previous studies (42–44). Furthermore, the analysis of WES data demonstrated all patients with ACC were MSI negative, and 17 of them were confirmed by routine MSI-PCR testing (**Figure 2B**).

In 75 patients with primary or recurrent malignant neoplasms who underwent surgical treatment, 62 tumor tissues were analyzed for PD-L1 expression by immunohistochemistry, and the presence of CD8^+^ immune cells was detected. The remaining 13 tumors could not be evaluated because specimens had an inadequate number of tumor cells. The majority of cases (72.6%, 45/62) did not show any membranous expression of PD-L1 (**Figure 2C**, left panel). Only 17 cases (27.4%, 17/62) of ACC displayed components with mild intensity of PD-L1 staining, accounting for approximately 1% of the tumor cells (**Supplementary Figure S1B**). The presence of infiltrating CD8^+^ immune cells was evaluated in the tumor tissue and the surrounding stroma. In general, CD8+ immune cells in the entire cohort had a low degree of tumor infiltration. Merely 11 tumors (17.7%, 11/62) showed >10% CD8^+^ prevalence (**Figure 2C**, right panel), and 23 tumors (37.1%, 23/62) were <1% or none (**Supplementary Table 2**).

### Immune Infiltration of Tumor Microenvironment may be Used as an Indicator of Primary Tumor Recurrence

**Figure 3:**
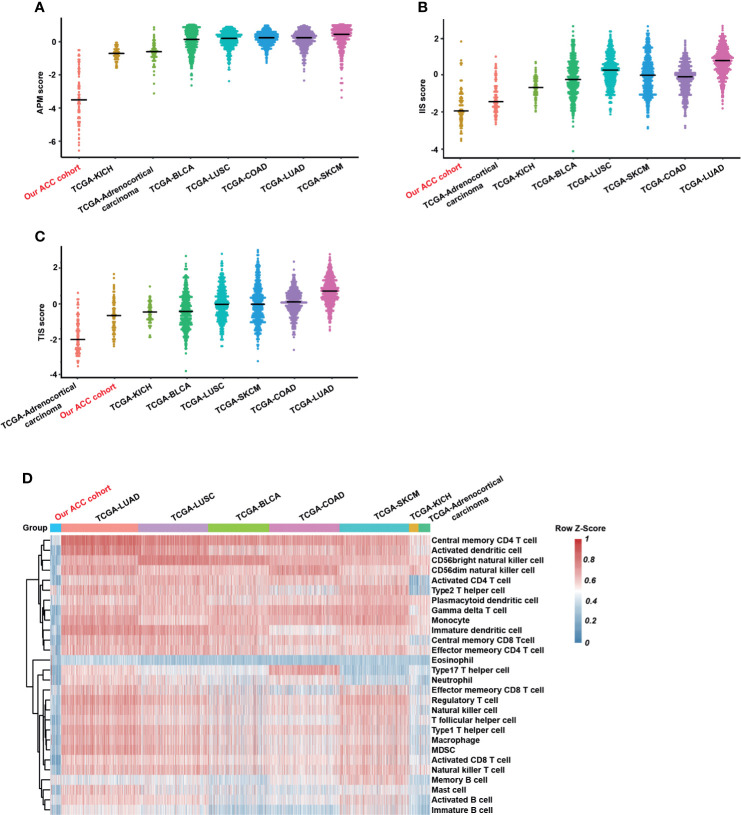
APM, immune infiltration, T cell infiltration, and immune cells in our ACC cohort compared with seven TCGA cohorts. **(A)** Antigen-presenting machinery (APM) score in eight tumor types. Each dot represents an individual tumor sample. Tumor types are ordered from left to right according to increasing median APM (medians indicated by horizontal black bars). **(B)** Overall immune infiltration score (IIS) for eight tumor types. Tumor types are ordered from left to right according to increasing median IIS (medians indicated by horizontal black bars). **(C)** T-cell infiltration score (TIS) in eight tumor types. Tumor types are ordered from left to right according to increasing median TIS (medians indicated by horizontal black bars). Immune cell Score estimated by ssGSEA for our cohort compared with adrenocortical carcinoma, BLCA, COAD, KICH, LUSC, LUAD, and SKCM TCGA samples, representing overall immune infiltration, based on gene level normalized count of respective RNA-Seq data. **(D)** Unsupervised clustering patients from our ACC cohort and 7 TCGA cohorts using ssGSEA scores from 28 immune cell types. ACC, adenoid cystic carcinoma; BLCA, urothelial bladder carcinoma; COAD, colon adenocarcinoma; KICH, kidney chromophobe; LUSC, lung squamous cell carcinoma; LUAD, lung adenocarcinoma; SKCM, skin cutaneous melanoma.

Şenbabaoğlu et al. employed mRNA-based scores for immune cell infiltration and the APM signatures, which were computed separately for each sample using ssGSEA (31). The TIS and IIS of each sample in the eight studied cancer types were calculated and used as the sum of the individual scores of the relevant immune subpopulations. Notably, the median APM score or IIS was the lowest in our cohort compared with seven other cancer types (**Figures 3A, B**), and the TIS of our ACC cohort was merely higher than that of TCGA adrenocortical carcinoma (**Figure 3C**). Furthermore, using the ssGSEA scores from the expanded panel of 28 immune-related and inflammation-related gene signatures, we observed that our ACC patients had low infiltration and weak CD8 signal compared with that of the samples of seven TCGA cancer types (**Figure 3D**). The low mutation load in ACC cells combined with the weak activity of antigen processing and presentation indicates that the availability of depressed tumor antigens may exist extensively in ACCs and foster an immune-poor tumor microenvironment.

**Figure 4:**
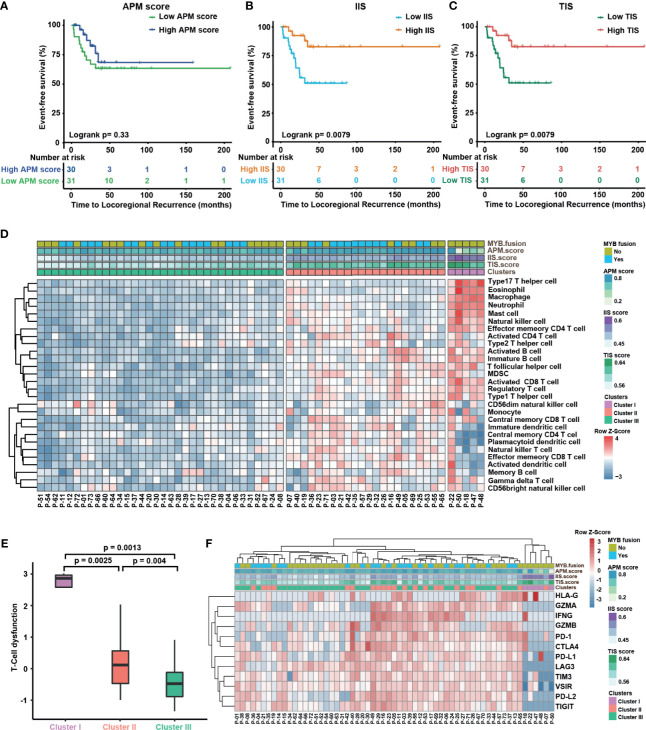
Immune infiltration of tumor microenvironment may be able to exert an indicator of primary tumor recurrence. **(A–C)** Kaplan–Meier curves for distant metastasis-free survival in the above-median and below-median groups for the APM score **(A)**, IIS **(B)**, and TIS **(C)**. The median values for IIS and TIS are able to stratify 65 primary cases into groups with significant locoregional recurrence differences. Statistical significance was assessed by using the log-rank test. **(D)** Unsupervised hierarchical clustering of ACC patients from 65 primary tumors using ssGSEA scores from 28 immune cell types. Hierarchical clustering was performed with Euclidean distance and Ward linkage. We discover three immune infiltration clusters, here termed (1) cluster I (2), cluster II, and (3) cluster III. **(E)** TIDE was used to analyze T-cell dysfunction among three clusters. Kruskal–Wallis test with Dunn correction (nonparametric). **(F)** Heat map showing expression of the inhibitory checkpoint molecules (PDCD1, PD-L1, PD-L2, LAG3, TIM3, CTLA-4, TIGIT, and VISTA) and effector molecules prominently associated with T-cell response (GZMA, GZMB, HLA-G and IFNG) across tumors from 65 primary cases.

These data lead us to explore whether the tumor microenvironment of primary disease is related to the ability of tumors to evolve locoregional recurrence or distant metastasis after initially definitive therapy. For 61 primary cases, the association of LRRFS or DMFS with APM score, IIS and TIS were analyzed by log-rank test, and the data suggested that APM gene expression alone was not associated with improved outcomes (**Figure 4A** and **Supplementary Figure S2F**), while ACCs with lower IIS (log-rank p = 0.0079) or TIS (log-rank p = 0.0079) score had shorter locoregional recurrence-free survival time (**Figures 4B, C**). However, IIS and TIS were not associated with DMFS (**Supplementary Figures S2G, H**), and the stage-specific differences in the expression levels of APM or IIS or TIS were not significant (**Supplementary Figures S2C–E**). To elucidate the underlying mechanism of immune cell infiltration on ACC recurrence, 61 primary cases were clustered into three clusters (cluster I, 5; cluster II, 22; and cluster III, 34) in terms of 28 immune cell types applied by unsupervised clustering. We observed that patients in the cluster II harbored higher APM scores, whereas cluster I have lower APM scores and higher IIS (**Figure 4D**, upper panel). Although there was no significant difference in clinical outcomes between patients in the cluster III and the other two clusters, a faster trend of recurrence was observed (**Supplementary Figures S3A, B**), which could be explained by a scarcity of immune surveillance in a poorly immune microenvironment. We further examined whether any T cells entered a state of dysfunctional or exhausted. A computational method to evaluate T-cell dysfunction, named TIDE, was utilized (38). Our results indicated significant differences in the level of T-cell dysfunction between the three clusters (**Figure 4E**). Furthermore, we analyzed the expression of inhibitory checkpoint molecules (PD-1, PD-L1, PD-L2, LAG3, TIM3, CTLA-4, TIGIT, and VISTA) and effector molecules (GZMB and IFNG) that were prominently associated with T-cell response. Strikingly, patients in the cluster I had lower expression of these inhibitory checkpoints compared to clusters II and III (**Figure 4F**). These results suggested T-cell dysfunction does not play a critical role in the recurrence of ACCs.

Furthermore, the prognostic significance of immune infiltration signatures and other clinicopathological features was examined using univariate and multivariate regression models (see **Table 2** and **Supplementary Table 2**). Factors related to a shorter LRRFS in univariate analysis were high IIS [hazard ratio (HR) = 0.32; 95% CI, 0.11–0.92; p = 0.035], high TIS (HR = 0.33; 95% CI, 0.12–0.94; p = 0.037), with postoperative radiotherapy (PORT, HR = 0.18; 95% CI, 0.06–0.51, p = 0.001), the presence of necrosis (HR=3.91, 95% CI 1.38 to 11.09, p= 0.01), and solid growth pattern (HR = 16.13; 95% CI, 5.80–44.87; p = 0.00). In the multivariate analysis, the solid growth pattern was shown to be an independent prognostic factor related to tumor recurrence (HR = 20.63; 95% CI, 4.35–97.75; p = 0.0001), and postoperative radiotherapy was also confirmed as an excellent independent prognostic factor (HR = 0.07; 95% CI, 0.02–0.31; p = 0.0004). Similarly, the solid growth pattern (HR = 3.4; 95% CI, 1.53–7.54, p = 0.003) and the relapsing disease (HR = 2.0; 95% CI, 1.03–4.01; p = 0.041) were confirmed to be correlated with a higher rate of distant metastasis, and postoperative radiotherapy (HR = 0.36; 95% CI, 0.14–0.94, p = 0.036) was an independent prognostic factor related to improved clinical outcome.

**Table 2:** Univariate and multivariate analyses of factors associated with time to locoregional recurrence and distant metastasis in ACC cohort (N=61).

Variables	Time to locoregional recurrence				Time to distant metastasis			
	Univariate P	Multivariate			Univariate P	Multivariate		
		HR	95%CI	P		HR	95%CI	P
Age, years (≥45 *vs.*<45)	0.653			NA	0.197			NA
Gender (male *vs.* female)	0.094			NA	0.441			NA
APM score (high *vs.* low)	0.301			NA	0.606			NA
IIS (high *vs.* low)	**0.035**	0.21	0.02-1.85	0.160	0.341			NA
TIS (high *vs.* low)	**0.037**	1.08	0.12-9.59	0.942	0.296			NA
TMB (high *vs.* low)	0.894			NA	0.465			NA
IndelTMB (high *vs.* low)	0.35			NA	0.904			NA
TNM stage (III-IV *vs.* I-II, unknown)	0.479			NA	0.252			NA
PORT (yes *vs.* no, unknown)	**0.001**	**0.07**	**0.02-0.31**	**0.0004**	**0.002**	**0.36**	**0.14-0.94**	**0.036**
PNI (positive *vs.* negative, unknown)	0.454			NA	0.384			NA
Necrosis (positive *vs.* negative, unknown)	**0.01**	1.66	0.46-5.97	0.435	0.656			NA
Margin (positive *vs.* negative, unknown)	0.826			NA	0.121			NA
Solid subtype (yes *vs.* no)	**0**	**20.63**	**4.35-97.75**	**0.0001**	**0.003**	2.01	0.65-6.25	0.228
*MYB* mutation (positive *vs.* negative)	0.156			NA	0.401			NA
State of disease (relapsed *vs.* naive)	NA	NA	NA	NA	**0.041**	0.98	0.38-2.51	0.961

Univariate analysis was calculated by the Kaplan–Meier method (log-rank test). Multivariate analysis was done using the Cox multivariate proportional hazard regression model with stepwise manner. Bolded values have statistic significance.

APM, antigen presenting machinery; IIS, immune infiltration score; TIS, T cell infiltration score; TMB, tumor mutational burden; Indel, insertions and deletions; TNM, tumor-nodes-metastases; PORT, postoperative radiotherapy; PNI, perineural invasion; HR, hazard ratio; CI, confidential interval; NA, not adopted.

In contrast, all parameters of immune infiltration were not significantly associated with DMFS in 14 cases of recurrent tumors (**Supplementary Figures S4A–C**). Unsupervised clustering of recurrent cases according to 28 immune cell types showed no predominantly separation, except for three cases with high immune infiltration (**Supplementary Figure S4D**). Unexpectedly, immune-rich cases had higher inhibitory checkpoints expression and APM score compared with other patients (**Supplementary Figures S4E, F**), slightly concordant with IFN-related gene expression (**Supplementary Figure S4G**). Although our results may indicated that immunotherapy is a potential choice for effective control of these characteristic recurrent tumors, no significant response was observed in patients with recurrent/metastatic ACC in recently updated trial (45).

### Baseline Elevation in Immune Infiltration Signature of ACC Patients Responding to PD-1 Blockade

**Figure 5:**
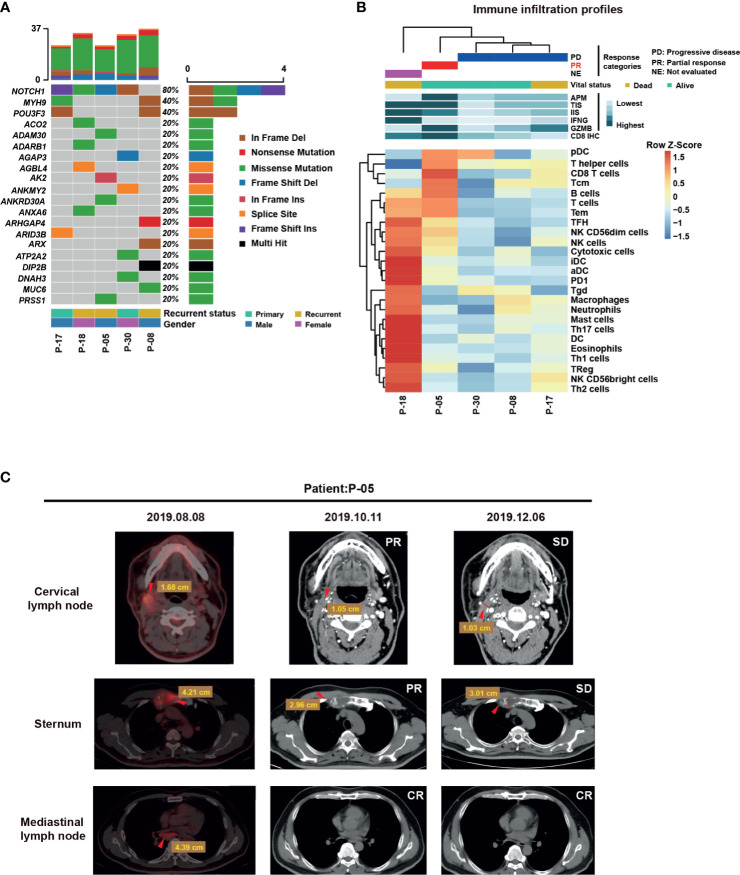
Mutational and Immune Infiltration Profiles in anti-PD1 inhibitor- treated ACC patients. **(A)** Data from combined WES sequencing of samples was analyzed to generate an oncoplot showing distribution of mutations. **(B)** RNA-Seq profiles of five ACC patients were generated and the patients were then treated with the anti-PD1 inhibitor pembrolizumab or camrelizumab. T cell infiltration as well as APM, TIS, and GZMB levels are generally high in a responder (partial response to camrelizumab). **(C)** Detection of recurrent metastatic ACC on positron-emission tomography-computed tomography (PET-CT) with ^18^ F-fluorodeoxyglucose (^18^ F-FDG) or CT. Images represent axial CT scans with volume reduction by RECIST criteria. The 53-year-old man (case No.P-05) in these images underwent immunotherapy and radiation therapy from August to December 2019. PET-CT was performed in August 2019. Axial views on PET (left panel) show disease recurrence with widespread metastases (red arrows). Contrast-enhanced CT was performed after the 4 cycles of immunotherapy in October 2019 and showed remarkable reduction in the volume of three metastases (middle panel, red arrows). Contrast-enhanced CT was performed after the 8 cycles of immunotherapy in December 2019 and showed stable disease (SD) (right panel, red arrows).

Given that we had determined the relationship between immune infiltration signatures and clinical status, we next investigated whether there was a correlation between the baseline immune landscape and response to immunotherapy. Since 2016, immunotherapy with anti-PD-1 inhibitors can be used to treat recurrent/metastatic squamous cell carcinomas of the head and neck. We investigated the pretreatment immune profile of patients treated with this agent using a set of five patients (**Table 3**). The mutational landscape of all five tumors revealed that *NOTCH1* mutations were prominent in this set (**Figure 5A**). Moreover, we found that the expression of APM, TIS, and GZMB was elevated in patients who partially responded to camrelizumab, whereas the expression was lower in patients with progressive disease with anti-PD-1 agents (**Figure 5B**). Remarkably, the responding patients displayed reduced tumor volume on all metastases (**Figure 5C**). This correlation should be corroborated in a larger cohort to determine whether it has predictive capability in determining the response to PD-1 blockade.

**Table 3:** Patients’ characteristics.

Nr	Sex	Age	Subtype	Stage	Anti-PD-1 Inhibitor	Line	Previous lines of therapy
P-18	F	60	Solid	Local recurrence, metastatic (bone)	Camrelizumab	1	
P-05	M	51	Solid	Local recurrence, metastatic (bone, mediastinal lymph nodes)	Camrelizumab	2	Paclitaxel,cisplatin
P-30	F	71	Unknown	Metastatic (lung, mediastinum, pleura)	Pembrolizumab	2	Apatinib
P-08	M	28	Mixed	Metastatic (lung)	Camrelizumab	4	Apatinib/Everolimus/Docetaxel, Nedaplatin
P-17	M	29	Mixed	Metastatic (lung, adrenal gland)	Camrelizumab	2	Apatinib

## Discussion

The treatment of ACC has not surpassed surgery and adjuvant radiotherapy in the past decades (8, 46), and no new drugs have been approved for the disease due to the limited understanding of the molecular alterations associated with aggressive disease (47). This comprehensive study of 75 ACCs provides certain novel insights into disease biology and delineates the immune microenvironment of tumors. Several genomic alterations identified in this study, particularly those involving the c-Myb and Notch1 pathways, have been fully confirmed for their potential oncogenic and prometastatic roles in ACC (48, 49). In particular, the Notch signaling blockade, AL101 showed clinical activity in recurrent/metastatic Notch mutant ACC and seems to be well tolerated (50). MYBL1 fusion in a subset of tumors lacking *MYB* alteration indicates that MYB-like signaling may be required for the development of ACC. In addition, we found mutations in genes encoding chromatin-state regulators, such as *KDM6A*, *CREBBP*, and *KMT2D*, which suggests that there is aberrant epigenetic regulation in ACC oncogenesis. 

TMB is an emerging independent biomarker of outcomes with immunotherapy for multiple tumor types (51–55). TMB with at least 10 mutations per megabase is an effective biomarker for lung cancer (51). The more mutations the tumor accumulates, the higher likelihood of production and subsequent presentation of neoantigens on major MHC molecules causing a higher tendency of tumor cell cytotoxicity after inhibition of checkpoint signals (56, 57). In our analysis, ACCs displayed an overall low mutation burden and had the lowest APM median, suggesting that antigen generation and presentation were in an inactive state, which may partially explain the low immune infiltration exhibited by ACC as a cold tumor (58). Additionally, we predicted that MHC-I would bind to tumor neo-antigens in patients receiving PD-1 blockade therapy (**Supplementary Methods**), while patients who responded to immunotherapy did not show high abundance of cancer neo-antigens (**Supplementary Figure S3C** and **Supplementary Table 3**). Some indels in the protein coding region may affect the structure or function of the protein (**Supplementary Table 4**). This potential finding should be corroborated in a larger cohort.

Three growth patterns of ACC have been described: cribriform, tubular, and solid. The cribriform and tubular growth patterns are less aggressive (4). Tumors that exhibit a solid pattern or have solid components are more likely to spread and have a worse prognosis (7–9). In our analysis, the solid growth pattern was shown to be an independent prognostic factor related to tumor recurrence (HR = 20.63; 95% CI, 4.35–97.75, p = 0.0001). Notably, our results validated that PORT may exert its beneficial effect by preventing locoregional recurrence and distant metastasis (**Table 2**).

Although the relationship between survival rate and benefit is still controversial (59–61), multiple studies have reported that the local control with PORT is better, regardless of the tumor stage (46, 62). This difference in clinical outcome is probably due to the effect of radiotherapy on TME. Of note, complicated immune responses to irradiated TME are neither utterly immunostimulatory nor immunosuppressive. These reactions involve effects on the cells inherent in TME such as altered in inflammatory cytokine production, antigen presentation, and dendritic cells (DC) priming, and relative expansion in radioresistant immunosuppressive macrophage and T-cell populations (63). A certain degree of PORT-induced immune response in TME may be predicted based on the baseline information of the primary lesion; it may be worth exploring adjusting immunotherapy strategy to overcome the adaptive immune suppression.

Our results highlighted that ACC tumors have the lowest median APM score and immune infiltration median (**Figures 3A, B**). The expression of APM genes and IFN-related genes was investigated and characterized as a similar expression pattern in primary cases (**Supplementary Figures S2A, S3D**), which is consistent with the previous discovery that IFN-γ activated antigen presentation (64). Preliminary evidence from TME studies suggests that the ACC microenvironment has low immunogenicity, represented by low TIL and DC density (65, 66), which was associated with β-catenin/Wnt and PI3K pathways (67, 68). Moreover, immune tolerance phenotype tumors are also accompanied by low or no PD-L1 expression (69), as observed in our ACC cohort. These observations could be explained to some extent by Theelen et al., who reported that impaired IFN-γ response signal transduction in tumor cells may be related to the devoid of PD-L1 expression (70). Compared with the other two clusters, low PD-L2 expression was observed in cluster I (**Figure 4F**), which may be related to the inactive IFN-γ signaling pathway (**Supplementary Figure S3D**).

Unsupervised clustering of primary cases using immune infiltration levels revealed three clusters of infiltrated tumors; however, this classification did not show prognostic significance (**Supplementary Figures S3A, B**). On the contrary, IIS and TIS are significant immune predictors of locoregional recurrence. We speculated that due to intertumor heterogeneity of immune infiltration in cluster I (**Figure 4D**), principal component analysis (PCA) on the ACC 28 immune cell types showed that a good difference between cluster II and III tumors (**Supplementary Figure S2B**). Mosconi et al. (65) have reported that the majority of ACC cases in their cohort would be classified as immune tolerance type (71). Even though ACC tumors exhibited supressed immune infiltration and T-cell responsiveness, IIS and TIS may still be indicators of disease recurrence. Coincidentally, equivalent results of univariate analysis of locoregional recurrence were obtained through IIS and TIS grouping (**Figures 4B, C**).

In our cohort, primary tumors are predominantly enriched for cluster II or III; however, cluster III patients showed increased propensity to relapse (**Supplementary Figure S3A**). There are similar observations in other tumor types (72–75). Strikingly, MYB fusion along with poor antigen-presenting function and the presence of a highly exhausted T cell, but low levels of inhibitory checkpoints, were observed in cluster I tumors. Although dysfunctional T cells have been exhibited to upregulate inhibitory receptors in multiple studies (76–78), comparable results have not yet observed in ACCs (**Figures 4E, F**). In other words, T-cell dysfunction may not be a distinguished indicator of ACC immunotherapy. Therefore, our data indicate that cluster III patients may respond poorly to T-cell checkpoint inhibitors due to reduced T-cell infiltration. In contrast, due to the relatively high level of inhibitory checkpoint molecules and APM score, cluster II patients could deserve to be further studied to evaluate possible immunotherapeutic strategies.

It should be noted that, although diverse clustering patterns were analyzed in recurrent disease cohort, we did not observe clear associations among APM, IIS, TIS, and clinical outcome (**Supplementary Figures S4A–C**). The intricacy of immune cell populations infiltrating tumors with their synergistic or opposing effects may affect tumors differently depending on their histological and molecular type, their stage, the microenvironment of the organ in which they plant, or the nature of the primary tumor or its metastases (79). Numerous researches have shown that strong lymphocyte infiltration was associated with improved clinical outcome in multiple tumor types (80–83), but in this case, immune evasion occurs perhaps due to the fact that T cell could not migrate to the tumor site.

The optimal immunotherapy for ACC has not yet been fully established. To date, the activity of multiple checkpoint inhibitors in ACC is currently under active investigation (ClinicalTrials.gov identifier: NCT03146650 and NCT04209660). In completed studies, the NISCAHN study showed that single-agent nivolumab had limited efficacy in patients with recurrent/metastatic ACC (45). Schoenfeld et al. presented a randomized phase II study of pembrolizumab with or without hypofractionated radiation; no objective response was observed in this study, but over half of ACCs achieved disease stability (84). Our anti-PD-1 mAb treatment response results indicate that monotherapy may not be an optimal choice for patients with ACC, especially those with poor antigen presentation. In addition, we analyzed the differential genes of patients with partial response (PR) to immunotherapy compared with progressive disease (PD). These genes have been analyzed through Connectivity Map (CMap) to output 10 drugs, of which chorambucil and altretamine are chemotherapy drugs with similar efficacy with PD-1 inhibitors (**Supplementary Figure S3E**). Preliminary studies have shown that tumor immune evasion can occur through high expression of PD-L1 or tumor immune infiltration of PD-1-positive T lymphocytes (85). Although PD-L1 expression was absent in most ACC cases reported here, immune infiltration plays an important role in TME and can be manipulated as a mechanism of immune surveillance. We have observed that patients with relatively lower dewelIIS and TIS recurred faster. It is noteworthy that the responding patient may be due to the existence of a clinically significant abscopal effect by combining radiotherapy (**Supplementary Figure S3F**). Checkpoint inhibitors administered before or concomitant with radiotherapy may be a worthwhile therapeutic strategy to be further explored in ACCs.

Several caveats limit the generalizability of our works. Given the rarity of ACC and the risks associated with tissue collection, we were limited by the absence of matched adjacent normal tissue that could be quantified for various immune markers and mRNA expression, despite retrieving samples collected over decades.

In the current study, we performed a comprehensive evaluation of the genomic characteristics and immune microenvironment of ACC. Collectively, ACC is a cancer type that is slightly immune infiltrated, which may partially explain the poor response to immunotherapy. On the other hand, immune infiltration may facilitate immune surveillance and act as an indicator of primary tumor recurrence. This analysis hints that immune infiltration patterns may act as potential biomarkers of immune therapy and may guide the development of novel drug combination strategies.

## Data Availability Statement

The datasets presented in this study can be found in online repositories. The names of the repository/repositories and accession number(s) can be found in the article/**Supplementary Material**.

## Ethics Statement

The studies involving human participants were reviewed and approved by the Ethics Committee of Shanghai Ninth People’s Hospital (approval number: 2016-74-T31). A single-center database project involving head and neck cancer was approved in 2020 (approval number: SH9H-2020-T58-1). The patients/participants provided their written informed consent to participate in this study. Written informed consent was obtained from the individual(s) for the publication of any potentially identifiable images or data included in this article.

## Author Contributions

GPZ and DC designed the project. SJD, RRL, NH, YDY, and GDZ contributed to development of methodology. GPZ, SJD, RRL, WJ, LLY, and JL performed the patient treatment and collected the ACC samples and clinical information. SJD, NH, and DC processed and interpreted the data. SJD, DC, MZ, and GPZ wrote and revised the manuscript. YDY and YNY provided administrative, technical, and material support. All authors contributed to the article and approved the submitted version.

## Funding

This work was supported by grants from the Clinical Research Program of 9th People’s Hospital, Shanghai Jiao Tong University School of Medicine (JYLJ201825) and National Natural Science Foundation of China (31800700).

## Conflict of Interest

DC, NH, MZ, YNY, GDZ, and YDY were employed by the company GloriousMed Technology Co., Ltd.

The remaining authors declare that the research was conducted in the absence of any commercial or financial relationships that could be construed as a potential conflict of interest.

## Publisher’s Note

All claims expressed in this article are solely those of the authors and do not necessarily represent those of their affiliated organizations, or those of the publisher, the editors and the reviewers. Any product that may be evaluated in this article, or claim that may be made by its manufacturer, is not guaranteed or endorsed by the publisher.
