# Supplementary material for: The Immune Landscape of Chinese Head and Neck Adenoid Cystic Carcinoma and Clinical Implication
Source: Front Immunol. 2021 Sep 6;12:618367. doi: 10.3389/fimmu.2021.618367 (PMC8450584; doi:10.3389/fimmu.2021.618367)
Supplement: Supplementary file 5 [file DataSheet_5.docx]

**Supplementary Materials**

**The immune landscape of Chinese head and neck adenoid cystic carcinoma and clinical implication**

Shengjin Dou, MD^1,3,4†^, Rongrong Li, MD, PhD ^1,3,4†^, Ning He, MD^2^, Menghuan Zhang, PhD^2^, Wen Jiang, MD^1,3,4^, Lulu Ye, MD^1,3,4^,Yining Yang, PhD^2^, Guodong Zhao, PhD^2^, Yadong Yang, PhD ^2^, Jiang Li, MD,PhD^5^, Di Chen, PhD ^2*^, Guopei Zhu, MD^1,3,4*^

^1^Radiotherapy Division, Department of Oral and Maxillofacial-Head Neck Oncology, Shanghai Ninth People's Hospital, College of Stomatology, Shanghai Jiao Tong University School of Medicine, Shanghai, China.

^2^GloriousMed Technology Co., Ltd, Shanghai, China.

^3^National Clinical Research Center for Oral Diseases, Shanghai, China.

^4^Shanghai Key Laboratory of Stomatology & Shanghai Research Institute of

Stomatology, Shanghai, China.

^5^Department of Oral Pathology, Shanghai Ninth People's Hospital, College of Stomatology, Shanghai Jiao Tong University School of Medicine, Shanghai, China.

***Corresponding authors:**

Guopei Zhu, MD. E-mail: antica@gmail.com, Radiotherapy Division, Department of Oral and Maxillofacial-Head Neck Oncology, Shanghai Ninth People's Hospital, College of Stomatology, Shanghai Jiao Tong University School of Medicine, 639 Zhizaoju Road, Shanghai, 200011, P. R. China. Tel: +86 13774338182.

Di Chen, PhD. E-mail: nsadly1989@qq.com, GloriousMed Technology Co., Ltd, No.11, Lane 100, Banxia Road, Pudong New Area, Shanghai, 200011, P. R. China. Tel：+86(21)-38001188

*†These authors have contributed equally to this work*

This file contains 4 Figures and supplementary methods.

**Supplementary Figure S1**


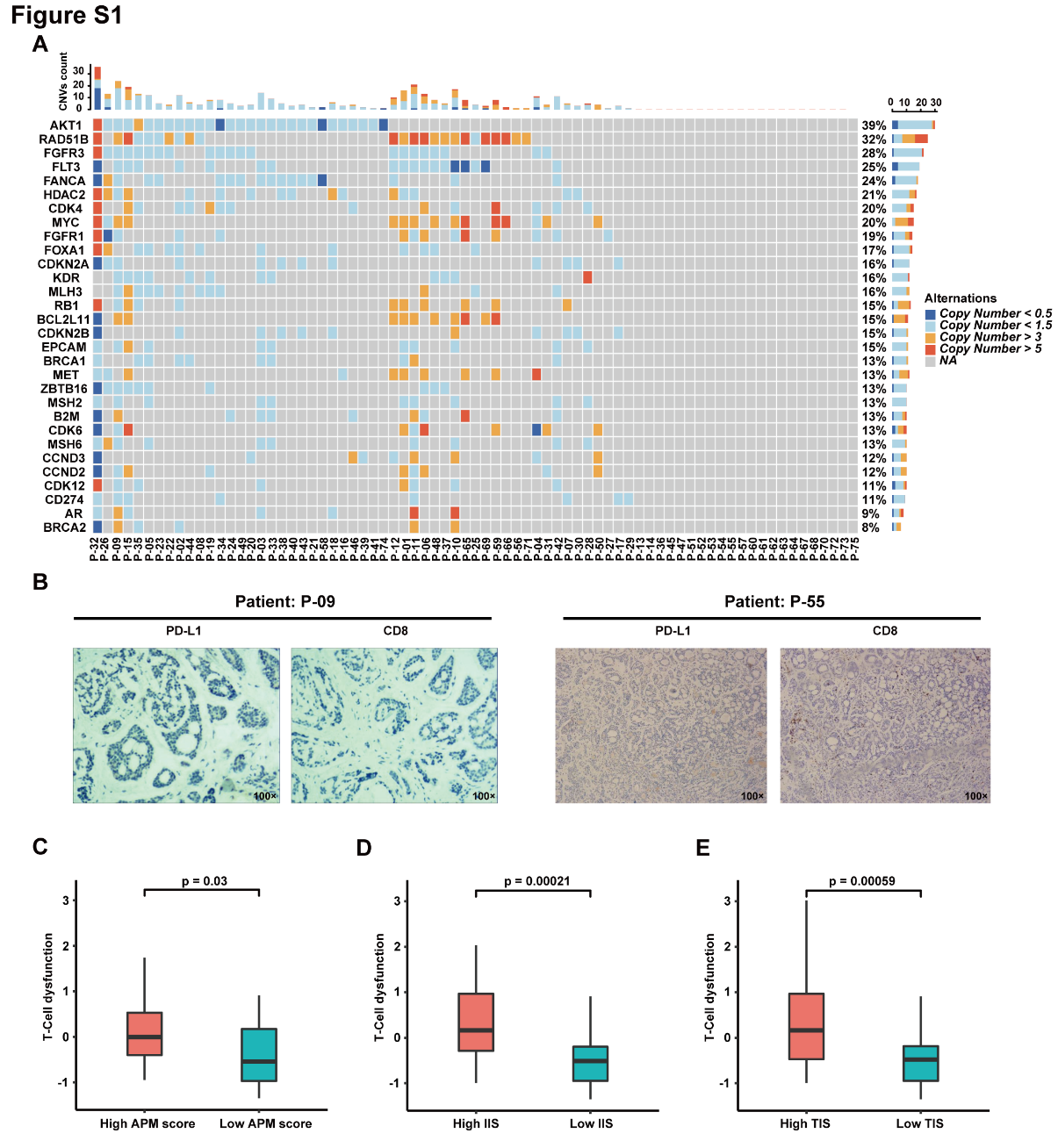


**Supplementary Figure S1. ACC as a tumor with a quiet genome and slightly immune infiltrated.**

**(A)** Deletions and amplifications for genomic regions with statistically significant focal copy number changes. ‘Copy number’ refers to absolute copy number. **(B)** Representative images for PD-L1 and CD8 immunohistochemical staining from case No.P-09 at 100× original magnification (left panel). Representative images for PD-L1 and CD8 immunohistochemical staining from case No.P-55 at 100× original magnification (right panel). **(C-E)** TIDE was used to analyze T-cell dysfunction in the above-median and below-median groups for the APM score (**C**), IIS (**D**) and TIS (**E**). Kruskal-Wallis test with Dunn correction (nonparametric).

**Supplementary Figure S2**


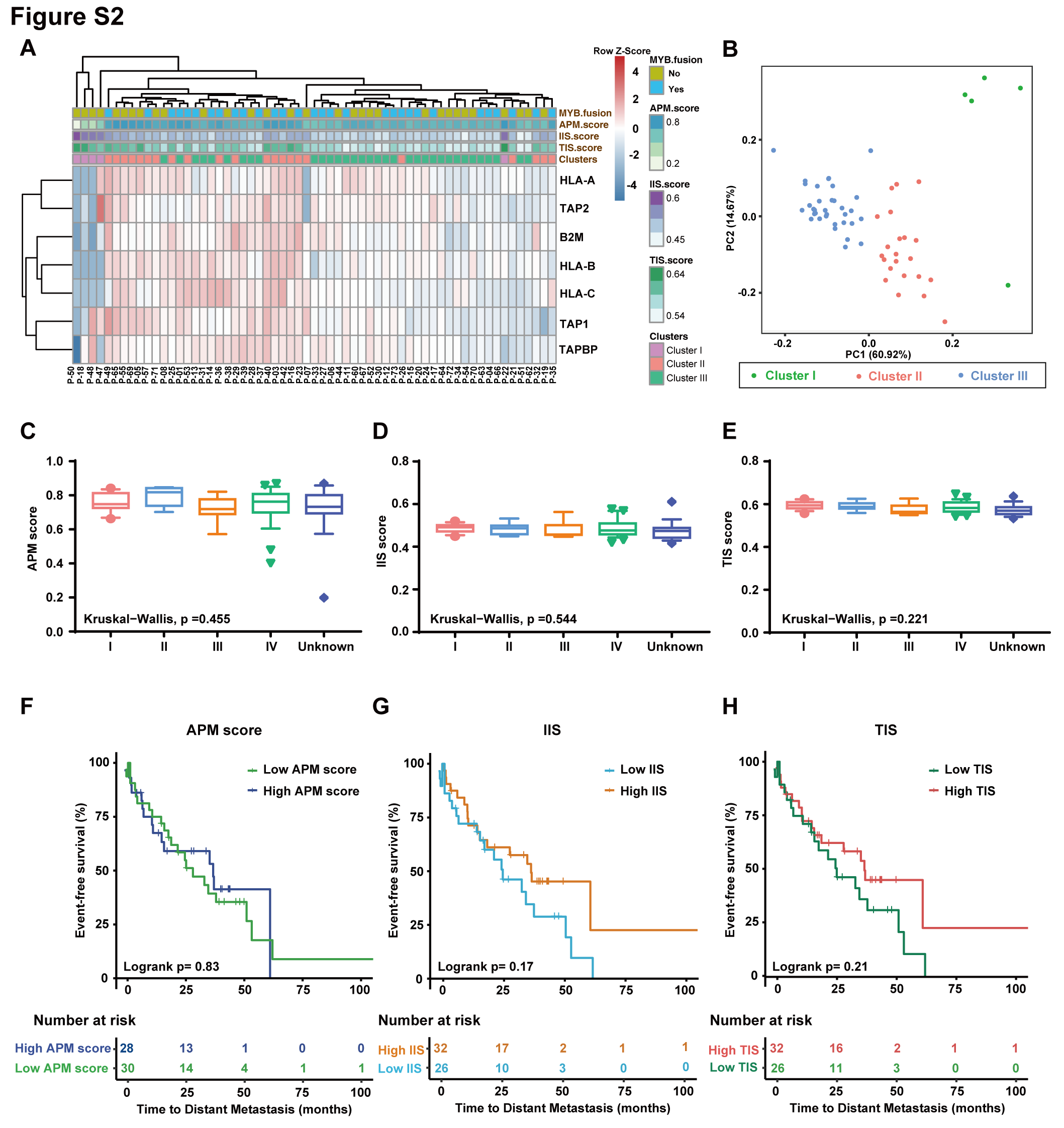


**Supplementary Figure S2. APM** **score, IIS and TIS are not predictive factors for distant metastasis.**

**(A)** Heat map showing expression of antigen presenting machinery (APM)-related gene across tumors from 65 primary cases. **(B)** Principal component analysis (PCA) of the 28 immune cell types in 65 primary cases. **(C)** Box and whisker blots are used to indicate the APM score for each stage group. **(D)** Box and whisker blots are used to indicate immune infiltration score (IIS) for each stage group. **(E)** Box and whisker blots are used to indicate T cell infiltration score (TIS) for each stage group. Each box represents the range from the first quartile to the third quartile. The median is indicated by a line. The whiskers outside the boxes represent the ranges from the minimum to the maximum value of each group. **(F-H)** Kaplan-Meier curves for distant metastasis-free survival in 65 primary cases. Patients were categorized as having APM score (**F**) or IIS (**G**) or TIS (**H**) above-median and below-median value group. Statistical significance was assessed by using the log-rank test.

**Supplementary Figure S3**


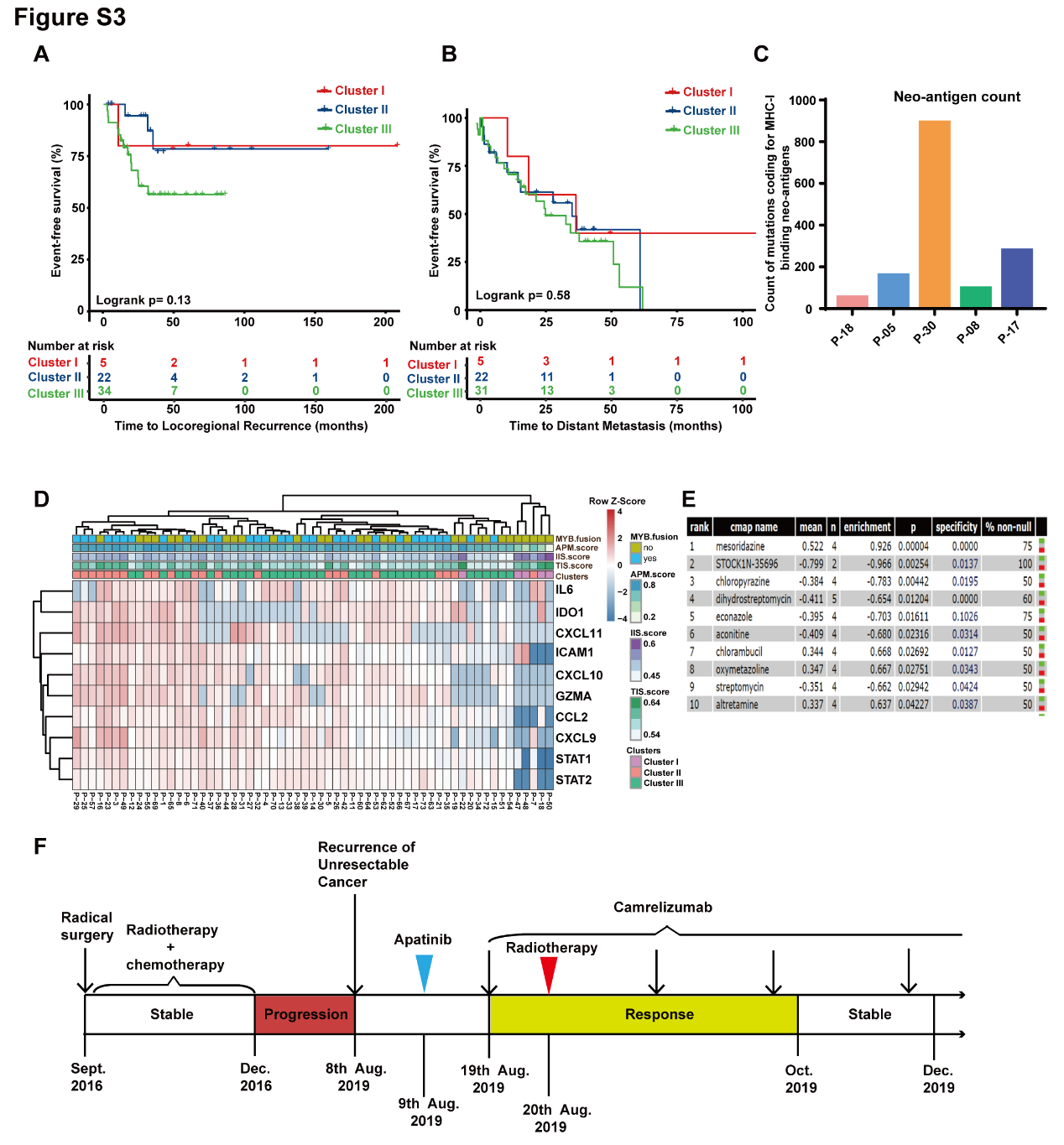


**Supplementary Figure S3. (A)** Kaplan-Meier curves for locoregional recurrence-free survival in 3 clusters. **(B)** Kaplan-Meier curves for distant metastasis-free survival in 3 clusters. Statistical significance was assessed by using the log-rank test. **(C)** The count of mutations that code for neo-antigen predicted in 5 patients treated with anti-PD1 inhibitors. **(D)** Heat map showing expression of a subset of genes from type II interferon (IFNγ) gene expression signature across tumors from 65 primary cases. **(E)** Top compounds that affect the expression of the gene expression signature genes in a manner similar to PD-1 inhibitors sorted by connectivity score. **(F)** The disease course of case No.P-05.

**Supplementary Figure S4**


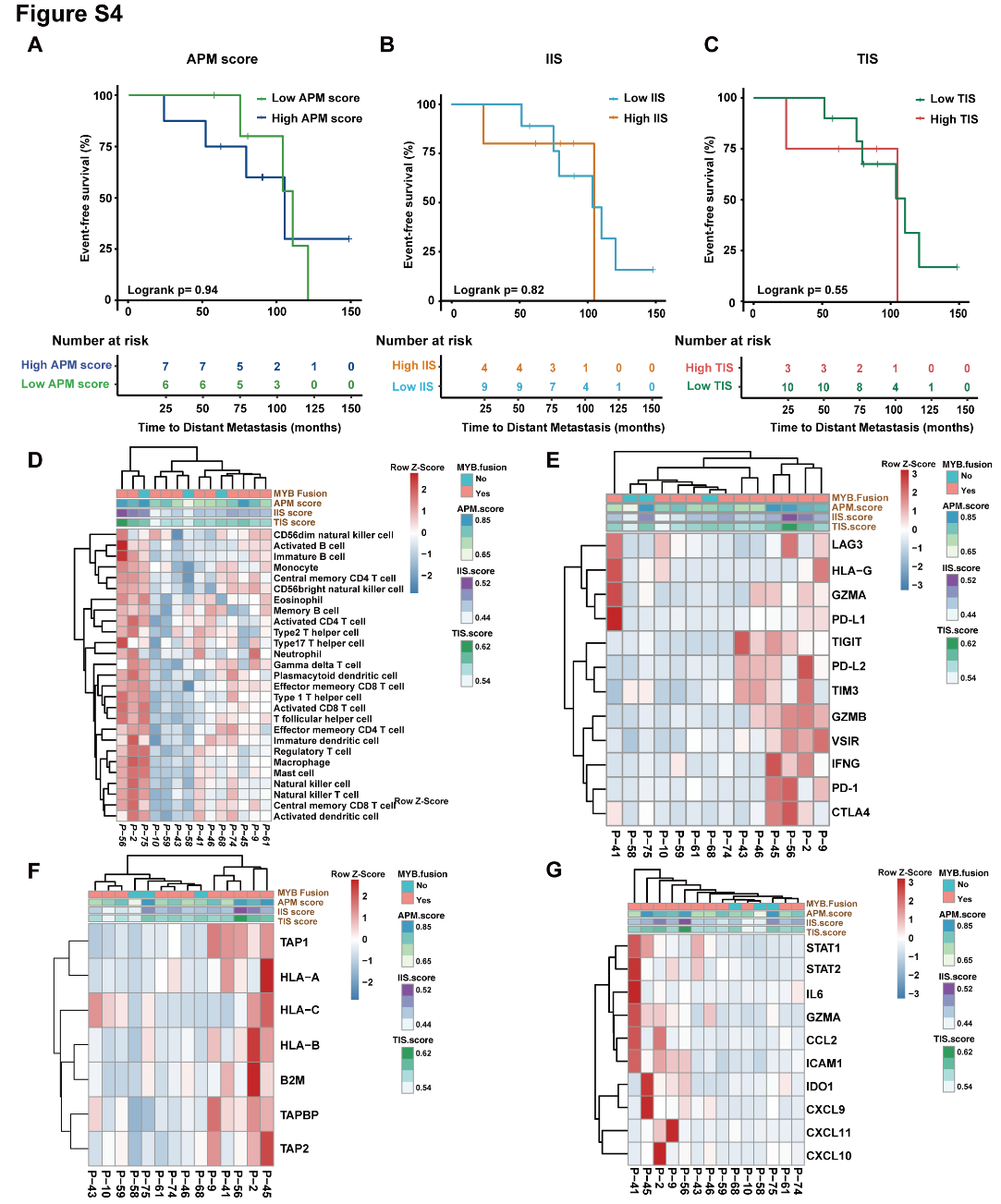


**Supplementary Figure S4. (A-C)** Kaplan-Meier curves for distant metastasis-free survival in 14 recurrent cases. Patients were categorized as having APM score (**A**) or IIS (**B**) or TIS (**C**) above-median and below-median value group. Statistical significance was assessed by using the log-rank test. **(D)** Unsupervised hierarchical clustering of 14 recurrent tumors using ssGSEA scores from 28 immune cell types. Hierarchical clustering was performed with Euclidean distance and Ward linkage. **(E)** Heat map showing expression of the inhibitory checkpoint molecules (PD-1, PD-L1, PD-L2, LAG3, TIM3, CTLA-4, TIGIT and VISTA) and effector molecules prominently associated with T cell response (GZMA, GZMB, HLA-G and IFNG) across 14 recurrent tumors. **(F)** Heat map showing expression of antigen presenting machinery (APM)-related gene across tumors from 14 recurrent cases. **(G)** Heat map showing expression of a subset of genes from type II interferon (IFNγ) gene expression signature across tumors from 14 recurrent cases.

**Supplementary methods**

***Quality Control and Variant Calling***

Sequencing adapters were trimmed by Trimmomatic([1](#_ENREF_1)) from the raw data. The reads after adapter trimming were then aligned with the human reference genome(hg19) by BWA([2](#_ENREF_2)). Duplicated reads were removed by Picard (http://broadinstitute.github.io/picard/). Mapped reads were also realigned to the genome by Genome Analysis Tool Kit([3](#_ENREF_3)). Somatic mutations and germline mutations were called by Mutect2 and GATK’s HaplotypeCaller with a paired workflow and GATK respectively([3](#_ENREF_3)). Variants were then annotated by ANNOVAR([4](#_ENREF_4)) and self-development code. An in-house script was used to verify the human identity concordance of paired samples. Somatic copy number alterations were also detected by GATK.

***MSI-PCR***

The microsatellite status of 17 tumor samples was determined using the Promega MSI analysis system (version 1.2, Promega, Madison, WI, US) following the manufacturer's instructions, and the PCR results were analyzed using GeneMapper v4.1 (Applied Biosystems, Foster City, CA, USA) software.

***Total RNA isolation***

Total RNA was isolated from each individual sample (53 tumor samples) using Qiagen RNeasy formalin-fixed paraffin-embedded (FFPE) Kit (Qiagen, Hilden, Germany) following the protocol supplied by the manufacturer. Purity and quantity of total RNA were measured by using Nanodrop equipment. Integrity of RNA was assessed using the RNA Nano6000 Assay Kit of the Bioanalyzer 2100 system (Agilent Technologies, CA, USA).

***RNA library preparation and sequencing***

A total amount of 1μg RNA per sample was used as input material for the RNA sample preparations. Strand-specific RNA sequencing libraries were generated using the Whole RNA-seq Lib Prep kit for Illumina (ABclonal, China) following manufacturer’s recommendations. Library quality was assessed on the Agilent Bioanalyzer 2100 system (Agilent, USA). Final libraries were sequenced at the Novogene Bioinformatics Institute (Beijing, China) on an Illumina Hiseq X10 platform by a 150bp paired-end reads.

***Target Capture and Sequencing***

Tumor FFPE DNA was extracted from 5 to 10 sections (5 mm thick) using the QIAamp DNA FFPE Tissue Kit (Qiagen, Hilden, Germany). Genomic DNA (gDNA) was extracted from white blood cells using the Blood Genomic DNA Mini Kit (Cwbiotech, China). xGen Exome Research Panel v1.0 (IDT, USA) was used to capture whole exome genes of FFPE DNA. For each sample, 200ng to 500ng FFPE DNA or 500ng gDNA was then used for library preparation and quantification guided by KAPA Hyper Prep protocols (Wilmington, MA, USA). Pools of 4-6 libraries were used to hybridize to the capture panel with 16 hours at 65 degrees. Washing, recovering and amplification were done sequentially according the standard procedures of IDT. Libraries were than purified by AMPure XP (Beckman Coulter, USA) and quantified by Qubit™ dsDNA HS Assay Kit (Thermo Fisher Scientific, Cleveland, OH, USA). Final libraries were sequenced on Illumina Novoseq6000 (PE150) (Illumina, USA).

***HLA-binding neoepitope prediction***

HLA types of patients were predicted from the raw exome-sequencing data of tumor samples using the Athlates([5](#_ENREF_5)) (version 2014_04_26), which uses a normal germline bam ﬁle as input. Identiﬁed non-silent mutations (including SNPs and indel) from WES were used to generate a comprehensive list of peptides 9 amino acids in length with the mutated amino acid represented in each possible position using pVACtools([6](#_ENREF_6)). Predicted neoantigens in correlation analysis were identiﬁed as those with following criteria: (a) median mutant (MT) score < 500; (b) tumor DNA depth ≥ 4; (c) transcript expression ≥ 0.05; (d) median fold change ≥ 1.2; (e) median wild-type (WT) score ≥ 50; (f) normal variant allele fraction (VAF) ≤ 0.1; (g) tumor DNA VAF ≥ 0.01; (h) WT epitope sequence does not have the same 3 consecutive amino acids (Supplementary Table 3).

***CMap***

The differential genes of patients with partial response (PR) to immunotherapy compared with progressive disease (PD) were enquired into CMap([7](#_ENREF_7)) (https://portals.broadinstitute.org/cmap/).

**References**

1. A. M. Bolger, M. Lohse and B. Usadel. Trimmomatic: a flexible trimmer for Illumina sequence data. *Bioinformatics* (2014) 30(15): 2114-20. doi:10.1093/bioinformatics/btu170

2. H. Li and R. Durbin. Fast and accurate short read alignment with Burrows-Wheeler transform. *Bioinformatics* (2009) 25(14): 1754-60. doi:10.1093/bioinformatics/btp324

3. A. McKenna, M. Hanna, E. Banks, A. Sivachenko, K. Cibulskis, A. Kernytsky, et al. The Genome Analysis Toolkit: a MapReduce framework for analyzing next-generation DNA sequencing data. *Genome Res* (2010) 20(9): 1297-303. doi:10.1101/gr.107524.110

4. K. Wang, M. Li and H. Hakonarson. ANNOVAR: functional annotation of genetic variants from high-throughput sequencing data. *Nucleic Acids Res* (2010) 38(16): e164. doi:10.1093/nar/gkq603

5. C. Liu, X. Yang, B. Duffy, T. Mohanakumar, R. D. Mitra, M. C. Zody and J. D. Pfeifer. ATHLATES: accurate typing of human leukocyte antigen through exome sequencing. *Nucleic Acids Res* (2013) 41(14): e142. doi:10.1093/nar/gkt481

6. J. Hundal, S. Kiwala, J. McMichael, C. A. Miller, H. Xia, A. T. Wollam, et al. pVACtools: A Computational Toolkit to Identify and Visualize Cancer Neoantigens. *Cancer Immunol Res* (2020) 8(3): 409-420. doi:10.1158/2326-6066.CIR-19-0401

7. J. Lamb, E. D. Crawford, D. Peck, J. W. Modell, I. C. Blat, M. J. Wrobel, et al. The Connectivity Map: using gene-expression signatures to connect small molecules, genes, and disease. *Science* (2006) 313(5795): 1929-35. doi:10.1126/science.1132939
